# Association of response to TNF inhibitors in rheumatoid arthritis with quantitative trait loci for *CD40* and CD39

**DOI:** 10.1136/annrheumdis-2018-214877

**Published:** 2019-04-29

**Authors:** Athina Spiliopoulou, Marco Colombo, Darren Plant, Nisha Nair, Jing Cui, Marieke JH Coenen, Katsunori Ikari, Hisashi Yamanaka, Saedis Saevarsdottir, Leonid Padyukov, S Louis Bridges Jr., Robert P Kimberly, Yukinori Okada, Piet L CM van Riel, Gertjan Wolbink, Irene E van der Horst-Bruinsma, Niek de Vries, Paul P Tak, Koichiro Ohmura, Helena Canhão, Henk-Jan Guchelaar, Tom WJ Huizinga, Lindsey A Criswell, Soumya Raychaudhuri, Michael E Weinblatt, Anthony G Wilson, Xavier Mariette, John D Isaacs, Ann W Morgan, Costantino Pitzalis, Anne Barton, Paul McKeigue

**Affiliations:** 1 Usher Institute of Population Health Sciences and Informatics, University of Edinburgh, Edinburgh, UK; 2 MRC Institute of Genetics and Molecular Medicine, University of Edinburgh, Edinburgh, UK; 3 Arthritis Research UK Centre for Genetics and Genomics, Centre for Musculoskeletal Research, Manchester Academic Health Science Centre, University of Manchester, Manchester, UK; 4 NIHR Manchester Biomedical Research Centre, Manchester Academic Health Science Centre, Manchester University NHS Foundation Trust, Manchester, UK; 5 Division of Rheumatology, Department of Medicine, Brigham and Women's Hospital, Boston, Massachusetts, USA; 6 Department of Human Genetics, Radboud Institute for Health Sciences, Radboud University Medical Center, Nijmegen, The Netherlands; 7 Department of Orthopedic Surgery, Tokyo Women's Medical University, Tokyo, Japan; 8 The Centers of Research Excellence in Science and Technology, Japan Science and Technology Agency, Tokyo, Japan; 9 Department of Rheumatology, School of Medicine, Tokyo Women's Medical University, Tokyo, Japan; 10 Division of Rheumatology, Department of Medicine Solna, Karolinska Institutet and Karolinska University Hospital, Stockholm, Sweden; 11 Institute of Environmental Medicine, Karolinska Institutet, Stockholm, Sweden; 12 Division of Clinical Immunology and Rheumatology, University of Alabama at Birmingham, Birmingham, Alabama, USA; 13 Department of Statistical Genetics, Osaka University Graduate School of Medicine, Osaka, Japan; 14 Laboratory of Statistical Immunology, Immunology Frontier Research Center (WPI-IFReC), Osaka University, Suita, Japan; 15 Amsterdam Rheumatology and Immunology Centre, Reade, Amsterdam, The Netherlands; 16 Department of Rheumatology, VU University Medical Centre, Amsterdam University Medical Centres, Amsterdam, The Netherlands; 17 Department of Clinical Immunology and Rheumatology, Amsterdam University Medical Center, University of Amsterdam, Amsterdam, The Netherlands; 18 Department of Rheumatology and Clinical Immunology, Kyoto University Graduate School of Medicine, Kyoto, Japan; 19 CEDOC, EpiDoC Unit, NOVA Medical School, Universidade NOVA de Lisboa, Lisbon, Portugal; 20 Department of Clinical Pharmacy and Toxicology, Leiden University Medical Center, Leiden, The Netherlands; 21 Department of Rheumatology, Leiden University Medical Center, Leiden, The Netherlands; 22 Rosalind Russell / Ephraim P Engleman Rheumatology Research Center, University of California San Francisco, San Francisco, California, USA; 23 Center for Data Sciences, Harvard Medical School, Boston, Massachusetts, USA; 24 EULAR Centre of Excellence/UCD Centre for Arthritis Research, Conway Institute of Biomolecular and Biomedical Research, University College Dublin, Dublin, Ireland; 25 y Université Paris-Sud, INSERM UMR1184, Hôpitaux Universitaire Paris-Sud, AP-HP, Le Kremlin Bicêtre, Paris, France; 26 Institute of Cellular Medicine, Newcastle University, Newcastle upon Tyne, UK; 27 Musculoskeletal Unit, Newcastle upon Tyne Hospitals NHS Foundation Trust, Newcastle upon Tyne, UK; 28 School of Medicine, University of Leeds, Leeds, UK; 29 NIHR Leeds Biomedical Research Centre, Leeds Teaching Hospitals NHS Trust, Leeds, UK; 30 Centre for Experimental Medicine and Rheumatology, William Harvey Research Institute, Queen Mary University of London, London, UK

**Keywords:** rheumatoid arthritis, pharmacogenetics, anti-tnf

## Abstract

**Objectives:**

We sought to investigate whether genetic effects on response to TNF inhibitors (TNFi) in rheumatoid arthritis (RA) could be localised by considering known genetic susceptibility loci for relevant traits and to evaluate the usefulness of these genetic loci for stratifying drug response.

**Methods:**

We studied the relation of TNFi response, quantified by change in swollen joint counts (ΔSJC) and erythrocyte sedimentation rate (ΔESR) with locus-specific scores constructed from genome-wide assocation study summary statistics in 2938 genotyped individuals: 37 scores for RA; scores for 19 immune cell traits; scores for expression or methylation of 93 genes with previously reported associations between transcript level and drug response. Multivariate associations were evaluated in penalised regression models by cross-validation.

**Results:**

We detected a statistically significant association between ΔSJC and the RA score at the *CD40* locus (p=0.0004) and an inverse association between ΔSJC and the score for expression of CD39 on CD4 T cells (p=0.00005). A previously reported association between CD39 expression on regulatory T cells and response to methotrexate was in the opposite direction. In stratified analysis by concomitant methotrexate treatment, the inverse association was stronger in the combination therapy group and dissipated in the TNFi monotherapy group. Overall, ability to predict TNFi response from genotypic scores was limited, with models explaining less than 1% of phenotypic variance.

**Conclusions:**

The association with the CD39 trait is difficult to interpret because patients with RA are often prescribed TNFi after failing to respond to methotrexate. The CD39 and *CD40* pathways could be relevant for targeting drug therapy.

**Key messages**

**What is already known about this subject?**

- To date, no strong associations of individual genetic loci with response to tumour necrosis factor inhibitors (TNFi) in rheumatoid arthritis (RA) have been identified, despite recent large efforts based on conventional genome-wide association studies and a crowdsourcing initiative.

**What does this study add?**

- We introduced a new methodological approach for localising genetic effects by using genotypic risk scores based on known genetic loci for related traits and likely biomarkers.
- We identified two genetic loci strongly associated with TNFi response in RA and demonstrated that the genetic determinants of TNFi response are different to the known susceptibility loci for RA.

**How might this impact on clinical practice or future developments?**

- Measurements of expression of *CD40* and CD39 and their corresponding pathways could be relevant for targeting drug therapy in RA.
- Our new methodological approach could be useful for localising genetic effects in traits for which assembling large sample sizes is not feasible, such as drug response.

## Introduction

Biologic therapies have transformed the outlook for rheumatoid arthritis (RA). However, for the most commonly used class of agent, tumour necrosis factor inhibitors (TNFi), there is substantial variability in response to treatment among patients with RA.^1^ This has spurred efforts to discover predictors of response and more generally to understand how to subtype this heterogeneous disease to predict which therapies will work.^2 3^

Genome-wide association studies (GWAS) of response to TNFi have shown that common single nucleotide polymorphisms (SNPs) explain an estimated 40% and 50% of the variance of change in swollen joint counts (SJC) and erythrocyte sedimentation rate (ESR), respectively; however, no strong associations with individual SNPs have been detected.^4^ Thus, as with many complex phenotypes, the genetic architecture of response to TNFi is likely to be polygenic with many small genetic effects.^5^ In this situation, the sample size required to learn a predictive model is very large—up to 10 cases per variable^6^—and it may not be feasible to assemble such large sample sizes for studying response to a single drug or drug class.

It has been suggested that improving prediction of complex clinical outcomes may be possible by incorporating information about the genetics of relevant traits in the prediction model.^7 8^ One such approach is to use publicly available summary GWAS results of relevant traits to compute genotypic scores, which can then be used as variables (‘features’) from which to build predictive models. By harnessing the genetic profiles of intermediate traits, these scores aggregate the effects of individual SNPs into larger regional or whole-genome effects. Relevant traits can include diseases, biomarkers and gene transcription levels. For polygenic traits such as RA, for which multiple genetic susceptibility loci have been identified, we can construct locus-specific scores allowing us to examine the extent to which drug response is related to genetic heterogeneity of the disease.

In the current study, we incorporated available genetic information on susceptibility to RA,^9^ immune cell traits from a publicly available bioresource^10^ and expression or methylation of genes implicated in response to TNFi treatment in RA.^11^ The genotypic scores associated with these intermediate traits were then tested for association with response to TNFi; by reducing the number of hypotheses being explored, the thresholds for claiming statistical significance are relaxed, which could help identify useful predictors.

## Materials and methods

### Cohorts

We used a sample of 2938 individuals of European ancestry for whom complete clinical and GWAS data were available. This sample comprised individuals from a pre-existing international collaboration formed to study the genetics of response to TNFi agents^12^ and individuals recruited to the Biologics in Rheumatoid Arthritis Genetics and Genomics Study Syndicate (BRAGGSS) after 2013.^4^

Table 1 shows sample sizes, phenotypes and clinical variables for each of the data collections used in this study. All participants provided informed consent, and institutional review board/ethics approvals were in place as described in Cui *et al* 12 and Massey *et al*.^4^

**Table 1:** Sample information per cohort.

	BRAGGSS	DREAM	EIRA	ReAct	WTCCC	Other*	Total
Sample size	954	764	283	258	556	123	2938
Sex, female %	76	68	74	77	77	82	74
Concomitant non-biologic DMARD %	85	74	74	50	73	97	76
*TNF inihibitor*							
Adalimumab	416	441	47	258	64	29	1255
Certolizumab	34	0	0	0	0	0	34
Etanercept	293	66	97	0	246	19	721
Golimumab	17	0	0	0	0	0	17
Infliximab	194	138	139	0	246	75	792
*Baseline disease activity, mean (* *SD* *)*							
DAS28-ESR4	6.3 (1.0)	5.4 (1.2)	5.3 (1.2)	5.8 (1.0)	6.7 (0.9)	5.6 (1.1)	6.0 (1.2)
ESR	36.2 (26.2)	27.7 (21.3)	32.4 (22.9)	31.2 (21.4)	45.0 (28.8)	30.2 (21.5)	34.6 (25.4)
SJC	10.1 (6.1)	10.3 (5.5)	9.2 (6.0)	9.9 (5.1)	11.8 (6.4)	10.5 (6.3)	10.4 (6.0)
TJC	15.4 (7.3)	10.0 (7.4)	8.3 (6.0)	13.1 (6.5)	17.0 (7.3)	10.7 (6.3)	13.2 (7.8)
GHVAS	71.0 (19.2)	62.3 (22.1)	56.1 (23.3)	59.7 (20.8)	72.6 (18.1)	59.3 (23.9)	66.1 (21.4)
*6* *-* *month* *disease activity, mean (* *SD* *)*							
DAS28-ESR4	3.7 (1.6)	3.6 (1.3)	3.5 (1.4)	3.7 (1.4)	4.2 (1.5)	3.9 (1.6)	3.8 (1.5)
ESR	22.8 (22.0)	18.1 (16.9)	20.0 (17.3)	18.6 (17.0)	27.6 (24.9)	22.2 (19.7)	21.8 (20.7)
SJC	3.0 (4.0)	4.8 (4.4)	3.4 (3.8)	3.6 (3.6)	4.0 (4.7)	5.0 (4.9)	3.8 (4.3)
TJC	5.0 (6.3)	3.7 (4.6)	3.6 (4.9)	4.7 (5.5)	6.3 (6.6)	5.1 (6.1)	4.7 (5.8)
GHVAS	37.2 (25.2)	34.5 (21.7)	34.4 (25.2)	31.8 (25.9)	37.1 (25.0)	31.8 (26.6)	35.5 (24.5)

* ‘Other’ displays aggregate sample characteristics for collections with sample size <100

BRAGGSS, Biologics in Rheumatoid Arthritis Genetics and Genomics Study Syndicate; DAS, Disease Activity Score; DMARD, disease-modifying antirheumatic drug; DREAM, Dutch Rheumatoid Arthritis Monitoring Registry; EIRA, Swedish Epidemiological Investigation of Rheumatoid Arthritis; ESR, erythrocyte sedimentation rate; GHVAS, global health assessment rated on a visual analogue scale; ReAct, French Research in Active Rheumatoid Arthritis; SJC, swollen joint count; TJC, tender joint count; TNF, tumour necrosis factor; WTCCC, Wellcome Trust Case Control Consortium.

### Definition of response to TNFi treatment

In RA, response to treatment is quantified by change in the Disease Activity Score (DAS), which depends on four measurements: ESR, SJC, tender joint count (TJC) and patient global health assessment rated on a visual analogue scale (GHVAS). Previous work has shown that only the SJC and ESR measurements have evidence of non-zero heritability^4^ and correlate significantly with synovitis quantified by ultrasound or MRI.^13 14^ Since TNFi were developed to control synovitis, we used the two objective components of the DAS (ESR and SJC) as primary outcomes for evaluating genetic effects and the two subjective components (TJC and GHVAS) and the composite score (DAS28-ESR4) as secondary outcomes. For each outcome, a baseline measurement was taken before initiation of TNFi treatment, and a follow-up measurement was taken between 3 and 6 months after initiation of TNFi treatment. The measurements for each component were transformed in accordance to the DAS28-ESR4 formula (see online supplementary methods). Response was modelled as the difference between the baseline and the follow-up measurement.

10.1136/annrheumdis-2018-214877.supp1 Supplementary data

### Genotypic risk scores

We used the GENOSCORES platform (https://pm2.phs.ed.ac.uk/genoscores/) to compute genotypic risk scores for the intermediate traits. GENOSCORES is a database of published SNP to trait associations from a large number of well-powered GWAS, including GWAS of disease traits, biomarkers, gene expression and methylation. The database is accompanied by a software package implemented in R that can be used to compute genotypic risk scores and run downstream statistical analyses in cohorts with SNP data.

We queried the GENOSCORES database for genetic associations with RA risk,^9^ 149 heritable immune cell traits reported by Roederer *et al*,^10^ and whole-blood expression and methylation for 93 genes reported in a recent meta-analysis^11^ as differentially expressed before treatment between responder and non-responder patients with RA treated with TNFi. GWAS summary statistics reported by Westra *et al* 15 and Gusev *et al* 16 were used for expression quantitative trait loci (eQTLs). GWAS summary statistics reported by Gaunt *et al* 17 were used for methylation quantitative trait loci (mQTLs).

GWAS summary statistics for each intermediate trait were filtered at p value<$10^{-5}$. SNPs were then split into trait-associated regions, with regions defined as genomic loci containing at least one SNP with p value<$10^{-7}$. Only 19 of the immune cell traits had a corresponding trait-associated region. SNPs not assigned to a region were discarded. For each trait-associated region, a genotypic score was computed as a sum of SNP genotypes, g, weighted by the effect size estimates, β (log OR for binary traits, regression slope for quantitative traits) and adjusted for linkage disequilibrium. The regional score, $s_{itr}$, for an individual i, trait t and region r was computed as: $s_{itr}=\beta_{tr}^{T}R_{r}^{-1}g_{ir}$, where $R_{r}$ denotes the SNP–SNP correlation matrix in genomic region r.

Additional details about the GENOSCORES platform, the score computation and the specific regional scores used in this study are given in online supplementary materials (see online supplementary methods, online supplementary tables S1–S5, online supplementary figure S1).

### Predictive modelling

To evaluate genetic prediction of response to TNFi, we compared a model with clinical covariates only to a model with clinical covariates and genotypic scores for each type of intermediate trait. To avoid numerical instabilities, we removed highly correlated scores prior to fitting a model (see online supplementary methods). The number of filtered regional scores for each type of intermediate trait is shown in table 2.

**Table 2:** Prediction of response to TNFi using penalised regional genotypic scores for different types of intermediate traits

Intermediate trait type	No of regional scores	No of filtered scores	Prediction of ∆SJC (%)	Prediction of ∆ESR (%)
Rheumatoid arthritis	37	37	5.3 (0.26)	−1.6 (0)
Immune cell traits	508	470	−0.7 (0)	2.9 (0.17)
eQTLs	94	87	3.4 (0.16)	2.9 (0.17)
eQTLs and mQTLs	268	228	2.5 (0.11)	1.6 (0.09)

Prediction performance is quantified by the difference in test log-likelihood (in nats) between a model with clinical covariates and genotypic scores and a model with clinical covariates only and by the per cent of phenotypic variance explained (in parenthesis). Results from 10-fold cross-validation.

eQTL, expression quantitative trait loci; mQTL, methylation quantitative trait loci.ESR, erythrocyte sedimentation rate; SJC, swollen joint count; TNFi, tumour necrosis factor inhibitors;

We expected that only a subset of genotypic scores would be relevant for prediction of response to TNFi, and thus used a hierarchical shrinkage prior for the score coefficients. We implemented the prediction models in STAN^18^ using a horseshoe prior distribution and performed inference with Markov chain Monte Carlo sampling.^19 20^ To rank the importance of genotypic scores in a model, we applied projection predictive variable selection, an approach that projects posterior draws from the high-dimensional model to lower dimensional subspaces.^21^

We used a statistical model with the following clinical covariates: measurements for the four DAS components before initiation of TNFi treatment, gender, whether the patient was concomitantly treated with any non-biologic disease-modifying antirheumatic drugs (DMARDs), cohort (which is also a proxy for country), genotyping array and the 10 first principal components computed from the genotypic data of the full data set. We used individuals with complete measurements in the statistical analyses of each TNFi response outcome (see online supplementary table S6).

#### Evaluation of prediction

We used two measures to quantify improvement in prediction: the difference in log-likelihood between a model with clinical covariates and genotypic scores and a model with clinical covariates only (measured in natural log units (nats)); and the per cent of residual variance explained by the genotypic scores. Both measures were computed on the testing data from 10-fold cross-validation on the full data set.

For readers who prefer a frequentist interpretation, the asymptotic equivalence of model choice by cross-validation and Akaike’s information criterion (AIC)^22^ implies that a p value of 0.01 for comparison of nested models is equivalent to a difference in test log-likelihood of 2.3 nats (likelihood ratio of 10) for models differing by one extra parameter. The large sample size of this study means that small robust increments in predictive performance can be detected.

### Univariate associations

For models with a test log-likelihood difference of at least two nats, we further examined the univariate associations between genotypic scores and the TNFi response outcomes. We used the full data set to test the univariate association and included the same clinical covariates as in the multivariate prediction, in addition to a score.

For genotypic scores significantly associated with TNFi response at the Bonferroni-corrected p value threshold, we compared the estimated effects among groups receiving different TNFi agents. We considered etanercept, adalimumab and infliximab, where a large enough sample size was available. Additionally, we tested if the associations held when we adjusted for additional covariates: anticitrullinated protein antibodies (ACPA) status and smoking status. These covariates have been reported to influence TNFi response but were only available for a third of the samples, and thus were not included in the full models.

A diagram of the statistical analysis pipeline is given in online supplementary figure S2.

## Results

In the following sections, we focus on the results for the primary TNFi response outcomes. The results for secondary outcomes are discussed in the online supplementary results (see online supplementary table S7).

### RA genotypic scores

Prediction of response to TNFi as quantified by ΔSJC improved by including the regional genotypic scores for RA risk in a penalised regression model (table 2). The test log-likelihood increased by 5.3 nats, suggesting that some of the genetic drivers for RA are also influencing response to TNFi; however, the absolute improvement in prediction was small, with less than 1% of phenotypic variance being explained by the RA genotypic scores.

**Figure 1:**
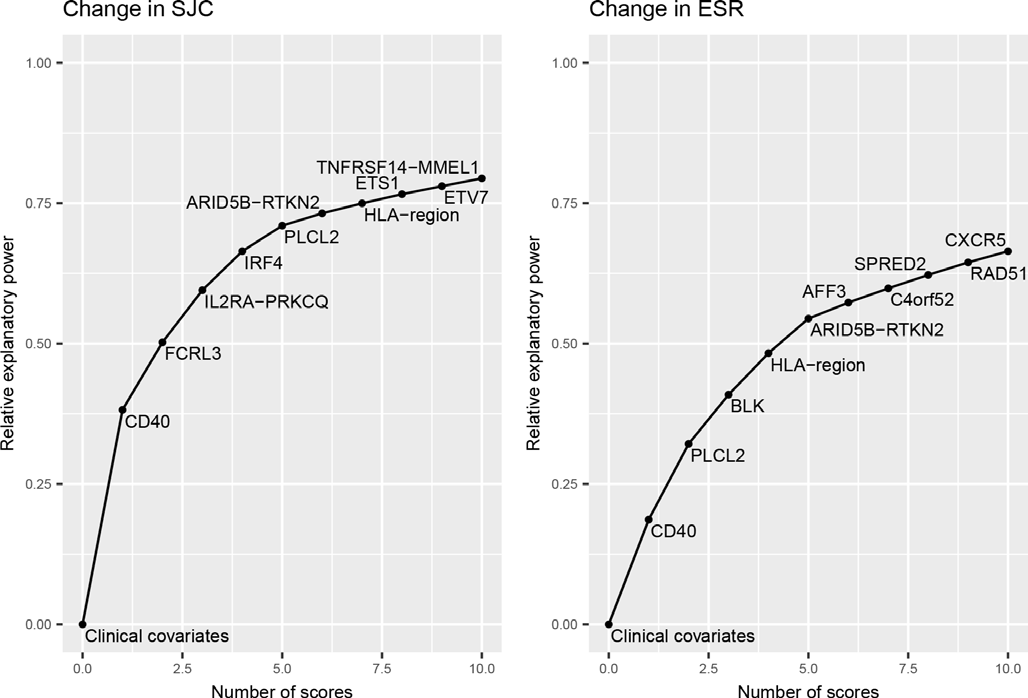
Contribution of top 10 RA regional scores to prediction of response to TNFi phenotypes, starting from a model containing only clinical covariates. The curve gradually converges to one with the addition of all remaining scores. ESR, erythrocyte sedimentation rate; RA, rheumatoid arthritis; SJC, swollen joint count; TNFi, tumour necrosis factor inhibitors.

The regional score at the *CD40* locus had the highest explanatory power for both response phenotypes (figure 1). The direction of the effect was consistent for response as measured by both phenotypes, with higher RA load at the *CD40* locus being associated with better TNFi response. The univariate association of the RA score at the *CD40* locus with ΔSJC passed the p value threshold corrected for the number of RA scores and two response phenotypes (table 3).

**Table 3:** Univariate associations between response phenotypes and regional genotypic scores of interest

Response phenotype	Genetic score	Coefficient	P value
*Genotypic scores at the CD40 locus*			
∆SJC	RA score at *CD40*	0.07	0.0004
∆SJC	*CD40* eQTL	0.06	0.002
∆SJC	*CD40* mQTL	−0.05	0.009
∆ESR	RA score at *CD40*	0.05	0.01
∆ESR	*CD40* eQTL	0.03	0.06
∆ESR	*CD40* mQTL	−0.03	0.07
*Genotypic scores for immune cell traits at the ENTPD1 locus*			
∆SJC	CD39 on CD4 T	−0.07	5e−05
∆SJC	mDC:%32+	−0.02	0.2
∆SJC	CD8:%39+	−0.06	0.001
∆SJC	CD4:%Treg(39+)	−0.07	0.0001
∆SJC	CD4:%Treg(39+73-)	−0.07	0.0001
∆SJC	CD4:%Treg(39+73+)	−0.07	0.0002
∆SJC	NKeff:%314−158a+	−0.04	0.02
∆SJC	CD4 T:%CD39+CD38+PD1−	−0.07	8e−05
∆ESR	CD39 on CD4 T	−0.003	0.9
∆ESR	mDC:%32+	−0.01	0.5
∆ESR	CD8:%39+	−0.008	0.7
∆ESR	CD4:%Treg(39+)	−0.004	0.8
∆ESR	CD4:%Treg(39+73−)	−0.004	0.8
∆ESR	CD4:%Treg(39+73+)	0.006	0.8
∆ESR	NKeff:%314−158a+	0.04	0.04
∆ESR	CD4 T:%CD39+CD38+PD1−	−0.004	0.8

The coefficients are the effect sizes of the standardised score on the standardised phenotype.

eQTL, expression quantitative trait loci; mTQL, methylation quantitative trait loci.ESR, erythrocyte sedimentation rate; RA, rheumatoid arthritis; SJC, swollen joint count;

The regional score for RA at the *CD40* locus was correlated with a cis-acting eQTL score for *CD40* expression in whole blood (correlation=0.65) and a cis-acting mQTL score for methylation of *CD40* in whole blood (correlation=−0.70). The strongest association between response to TNFi and genotypic scores at the *CD40* locus was with the score for RA (table 3). The estimated effect did not change when we stratified by TNFi agent and when we adjusted for ACPA and smoking status (see online supplementary tables S8 and S9).

### Genotypic scores for immune cell traits

Prediction of response to TNFi as measured by ΔESR improved by adding penalised genotypic scores for immune cell traits in the model (table 2). In univariate analyses, the regional scores for a number of immune cell traits at the *ENTPD1* locus (which codes for CD39) had suggestive associations with ΔSJC (table 3). The association of ΔSJC with the genotypic score for ‘CD39 on CD4 T cells’ at the *ENTPD1* locus passed the p value threshold corrected for the number of immune cell trait scores tested (470) and two response phenotypes. This score was correlated with the cis-acting eQTL score for *ENTPD1* (correlation=0.65). Higher score for ‘CD39 on CD4 T cells’ at the *ENTPD1* locus was associated with worse TNFi response as quantified by ΔSJC. There was no association between ΔESR and genotypic scores at the *ENTPD1* locus (table 3) and no association between either ΔESR or ΔSJC and a genotypic score for cell subset frequency of CD73, which is the second ectonucleotidase involved in adenosine production in regulatory T cells (see online supplementary results).

Previously, Peres *et al* 23 showed that low expression of CD39 on peripheral regulatory T cells was associated with worse response to methotrexate (MTX) in patients with RA. We note that the direction of the effect is reversed, but this is not unlikely since we considered response to TNFi. To further investigate the effect of ‘CD39 on CD4 T cells’ expression on TNFi response, we performed two analyses stratified by concomitant treatment. Information on whether a patient was receiving a concomitant non-biologic DMARD was available for all samples, while information on whether a patient was specifically receiving concomitant MTX treatment was available for a subset of patients from the BRAGGSS cohort. Table 4 shows the effect of the genotypic score for expression of ‘CD39 on CD4 T cells’ on ΔSJC for patients receiving TNFi treatment stratified by concomitant treatment with either any non-biologic DMARD (top) or specifically with MTX (bottom). The effect became stronger in the groups receiving concomitant treatment and attenuated in the group receiving TNFi monotherapy; the CIs among all groups overlapped. Similarly, we did not detect statistically significant differences when we stratified by TNFi agent and when we adjusted for ACPA status (see online supplementary tables S8 and S9).

**Table 4:** Univariate association between ∆SJC and genotypic score for the expression of ‘CD39 on CD4 T cells’ at the *ENTPD1* locus stratified by concomitant treatments

Patient group	Coefficient (SE)	P value	Sample size
All samples, adjusted for concomitant DMARD	−0.07 (0.02)	5e−05	2922
All samples, no adjustment for DMARD	−0.08 (0.02)	2e−05	2922
Samples not on concomitant DMARD	−0.02 (0.04)	0.5	691
Samples on concomitant DMARD	−0.09 (0.02)	1e−05	2231
Samples on concomitant MTX	−0.1 (0.03)	0.002	958

The coefficients are the effect sizes of the standardised score on the standardised phenotype. The phenotype is adjusted for covariates: baseline DAS components, gender, cohort, genotyping array, 10 genetic principal components.

DAS, Disease Activity Score; DMARD, disease-modifying antirheumatic drug; MTX, methotrexate; SJC, swollen joint count.

### eQTL and mQTL scores

Prediction of response to TNFi as quantified by both ΔSJC and ΔESR improved by adding eQTL and mQTL scores of implicated genes in a penalised regression model (table 2). Of the 93 genes reported in Kim *et al* 11 as differentially expressed between responders and non-responders to TNFi in RA, 54 genes had at least one eQTL, 54 genes had at least one mQTL and 36 had both. The test log-likelihood increased by 3.4 and 2.9 nats for ΔSJC and ΔESR, respectively, by adding the eQTL scores in the model. We did not see a further improvement by adding genotypic scores for mQTLs.

## Discussion

In the largest international study of TNFi response to date, we have shown how using methods that leverage information from relevant intermediate traits can identify predictors of TNFi response. In a recent crowdsourced effort to use machine learning to construct a predictor of response to TNFi in RA, including SNP genotypes did not improve prediction beyond that obtained with clinical covariates alone.^24^ Genotypic prediction of psychiatric disorders and related phenotypes has been shown to improve by exploiting genetic correlations among multiple related traits,^7 25^ and methods have been extended to incorporate polygenic scores for multiple traits.^8^

In the current study, we have combined these approaches and implemented them in a newly developed platform, called GENOSCORES, which contains GWAS data for multiple traits and automates construction of genotypic scores. For polygenic traits with multiple trait-associated loci, such as RA, locus-specific scores can be constructed to examine how genetic heterogeneity of the intermediate trait can influence the trait of interest. Our approach reduces the dimensionality of the prediction task from about 2 million common SNPs to a few hundred or a few thousand genotypic scores, depending on how relevant traits are selected. The score constructions are a type of feature engineering, a task commonly used in machine learning applications.

Understanding the pathogenic mechanisms that initiate and perpetuate RA could give rise to informative biomarkers of prognosis, therapeutic response and toxicity.^26^ However, in agreement with earlier studies,^27^ we did not find strong predictors of TNFi response among alleles linked to the development of RA. A strength of the current study is the large sample size which allowed us to detect small robust increments in predictive performance. Our methodological approach was to first establish the predictive value for a set of genetic markers using a multivariate model and then to examine univariate associations between each marker and the outcome. Using this approach, we showed that a model including RA scores led to a small robust improvement in prediction, with the regional score at the *CD40* locus driving the predictive signal.

Higher RA risk at the *CD40* locus, higher *CD40* transcription and lower *CD40* methylation were associated with better TNFi response. CD40 is a transmembrane protein which belongs to the TNF receptor superfamily, critically important in modulating immune-(auto-immune) responses.^28^ CD40 is expressed by B cells and antigen-presenting cells (APCs), whereas CD40L is induced on CD4+ T cells following T-cell antigen receptor (TCR) with major histocompatability complex (MHC) molecule interaction. Engagement of the CD40–CD40L axis leads to B cell activation, proliferation and (auto)-antibody production, while activation of APCs by CD40L on CD4+ T cells induces upregulation of CD80, CD86, MHC class I and MHC class II, as well as secretion of proinflammatory cytokines such as interleukin (IL)-12, IL-23 and TNF-α.^29 30^

The risk allele associated with RA is associated with elevated *CD40* expression in whole blood.^31^ As high *CD40* expression is associated with elevated TNF-α production and *CD40* and *CD40L* transcripts are increased in the disease tissue in both early and established disease,^32^ it is not surprising that patients with the *CD40* risk allele respond better to TNFi therapies.

Overall, if there are genetic loci that predict TNFi response, these are mostly different to the known RA risk loci. However, we note that patients who receive TNFi therapy are likely to have more severe disease and to have failed on other treatments. It is therefore possible that our study sample has been selected with respect to genetic load for RA, thus limiting the heterogeneity in genetic RA risk profiles compared with a sample of newly diagnosed cases.

The genotypic score for the expression of the ectonucleotidase CD39 on CD4 T cells was inversely associated with TNFi response. The SNPs contributing to this score are in the *ENTPD1* gene which encodes CD39. In stratified analyses, the inverse association with response was stronger in the groups receiving TNFi concomitantly with MTX or another non-biologic DMARD compared with the group receiving TNFi monotherapy and was stronger in the group receiving infliximab compared with the groups receiving adalimumab or etanercept. The CIs of the estimated effects among all groups overlapped. This effect on response to TNFi agents is in the opposite direction to the association reported between low expression of CD39 on regulatory T cells and resistance to MTX in RA.^23^

Interpreting the association between drug response and the CD39 trait is difficult both epidemiologically and mechanistically. The reasons for this being that in the UK and most European countries TNFi are usually prescribed only after patients have had a poor response to MTX and, unless of intolerance, always in combination with MTX. Therefore, these patients are likely to represent a selected group; this, together with the almost universal use of combination MTX/TNFi therapy, hinders progress in dissecting the potential mechanisms responsible for the divergence. Nonetheless, as RA is a highly heterogeneous disease, it is plausible to speculate that different cellular and molecular networks may be involved in driving diverse immune/inflammatory responses in different patients to different drugs. For example, as mentioned above, the poor response to MTX has been associated with low CD39 expression by regulatory T cells, while increased CD39 expression has been reported to be important in the expansion of Th17 cells driven by IL-6 and TGF-β via Stat3 and Gfi-1 transcription factors.^33^ In turn, the expansion of Th17 cells has been reported to be associated with incomplete response to TNFi.^34^ It remains to be established whether measurements of CD39/*ENTPD1* expression or genotype may be useful in the choice of MTX, TNFi agent or concomitant treatment as first-line therapy for patients who need a DMARD.

Using previously reported associations between transcripts and TNFi response to select relevant genes, we have shown evidence that eQTL scores for these genes contain information that predicts TNFi response, even though the proportion of variance explained was low and no single genes associated with response could be identified.

Improved genomic prediction of treatment response requires measuring response more precisely to capture the molecular in addition to the clinical phenotype. The detected associations between genotypic scores and TNFi response were not the same for the two measures of response—change in SJC and change in ESR—suggesting that the two measures reflect different aspects of disease activity affected by TNFi. To derive refined measures of drug response, large data sets with multiple inflammatory biomarkers, joint imaging and clinical variables before and after treatment are needed.

10.1136/annrheumdis-2018-214877.supp2 Supplementary data

10.1136/annrheumdis-2018-214877.supp3 Supplementary data

10.1136/annrheumdis-2018-214877.supp4 Supplementary data

10.1136/annrheumdis-2018-214877.supp5 Supplementary data

10.1136/annrheumdis-2018-214877.supp6 Supplementary data

10.1136/annrheumdis-2018-214877.supp7 Supplementary data
