# Supplementary material for: Association of response to TNF inhibitors in rheumatoid arthritis with quantitative trait loci for CD40 and CD39
Source: Ann Rheum Dis. 2019 Apr 29;78(8):1055–61. doi: 10.1136/annrheumdis-2018-214877 (PMC6669378; doi:10.1136/annrheumdis-2018-214877)
Supplement: Supplementary data [file annrheumdis-2018-214877supp001.pdf]

# Supplementary Information

Association of response to TNF inhibitors in rheumatoid arthritis with quantitative trait loci for *CD40* and CD39

## Contents


**1 Supplementary Methods** **3**
- 1.1 Definitions of TNFi response outcomes . . . . . . . . . . . . . . . . 3
- 1.2 Computation of genotypic principal components . . . . . . . . . 3
- 1.3 The GENOSCORES platform . . . . . . . . . . . . . . . . . . . . . 4
- 1.4 GENOSCORES score computation . . . . . . . . . . . . . . . . . 4
- 1.5 Genome-wide score for RA . . . . . . . . . . . . . . . . . . . . . . 5
- 1.6 Filtering of correlated scores . . . . . . . . . . . . . . . . . . . . . 5

**2 Supplementary Results** **5**
- 2.1 Prediction of secondary TNFi response outcomes . . . . . . . . . 5
- 2.2 Stratification by TNFi agent . . . . . . . . . . . . . . . . . . . . . 6
- 2.3 Adjustment for ACPA and smoking status . . . . . . . . . . . . . 6
- 2.4 Adjustment for genetic principal components with different LD-thinning . . . . . . . . . . . . . . . . . . . . . . . . . . . . . . . . 7
- 2.5 Association with CD73 genotypic score . . . . . . . . . . . . . . . 7

**3 Supplementary References** **7**


## List of Tables


- S1 Description of studies available in GENOSCORES. . . . . . . . . 8
- S2 RA regional scores: description and univariate associations. . . . 8
- S3 Immune cell traits regional scores: description and univariate associations. . . . . . . . . . . . . . . . . . . . . . . . . . . . . . 8
- S4 eQTL scores: description and univariate associations. . . . . . . . 8
- S5 mQTL scores: description and univariate associations. . . . . . . 8
- S6 Number of samples with complete measurements for each TNFi response outcome and two sets of clinical and lifestyle covariates. 8
- S7 Prediction of TNFi response outcomes from genotypic scores. . . 8
- S8 Univariate associations stratified by TNFi agent. . . . . . . . . . 9
- S9 Univariate associations after adjusting for different covariates . . 9
- S10 Univariate associations after adjusting for genetic principal components at different LD-thinning thresholds . . . . . . . . . . . . 9
- S11 Univariate associations with genotypic score for CD73. . . . . . . 10
- S12 MATURA collaborators and affiliations. . . . . . . . . . . . . . . . 10

**List of Figures**

S1 GENOSCORES flowchart demonstrating inputs, functionality and outputs. . . . . . . . . . . . . . . . . . . . . . . . . . . . . . . . 11
S2 Diagram of the statistical analysis pipeline. . . . . . . . . . . . . 12

## 1. Supplementary Methods

### *1.1. Definitions of TNFi response outcomes*

We used the natural logarithmic transformation for ESR and the square root transformation for SJC and TJC. These transformations reduce the right-skewness in the empirical distributions of the corresponding phenotypes. The square root transformation has a weaker effect compared to the logarithm, but it can also be applied to zero values, which can occur for SJC and TJC. These transformations are consistent with the computation of the DAS28-ESR4 composite score.

The distributions of the transformed phenotypes are nearly symmetric allowing us to make the convenient assumption of a normally distributed outcome in our statistical models. Although it is possible to use non-Gaussian distributions for model residuals, the normality assumption is convenient and simplifies inference. We note that the difference of two normally distributed variables is also normally distributed.

Overall, the TNFi response outcomes were defined as follows:

- $\Delta\text{ESR} = \ln(\text{ESR}_{\text{baseline}}) - \ln(\text{ESR}_{\text{follow-up}})$,
- $\Delta\text{SJC} = \sqrt{(\text{SJC}_{\text{baseline}})} - \sqrt{(\text{SJC}_{\text{follow-up}})}$,
- $\Delta\text{TJC} = \sqrt{(\text{TJC}_{\text{baseline}})} - \sqrt{(\text{TJC}_{\text{follow-up}})}$,
- $\Delta\text{GHVAS} = \text{GHVAS}_{\text{baseline}} - \text{GHVAS}_{\text{follow-up}}$,
- $\Delta\text{DAS28-ESR4} = \text{DAS28-ESR4}_{\text{baseline}} - \text{DAS28-ESR4}_{\text{follow-up}}$,

and the composite score was computed as:

- $\text{DAS28-ESR4} = 0.56 \times \sqrt{(\text{TJC})} + 0.28 \times \sqrt{(\text{SJC})} + 0.70 \times \ln(\text{ESR}) + 0.014 \times \text{GHVAS}$.

### *1.2. Computation of genotypic principal components*

We used the first 10 genetic principal components as covariates in all statistical models to account for population substructure due to varying allele frequencies in cohorts with different ancestries. The principal component analysis was performed in PLINK v1.9 [1] using common SNPs (minor allele frequency $>$ 0.05). To avoid any collinearity problems, we additionally thinned SNPs for linkage disequilibrium, setting the $r^2$ threshold for the pairwise correlation between SNPs to 0.9.

During the review process, it was pointed out that a lower threshold for LD thinning ($r^2$=0.2) would be preferable, to ensure that the PCs capture genome-wide genetic background, rather than genetic variation in regions with extended LD, such as the HLA. We evaluated whether the univariate association between the primary TNFi response outcomes and genotypic risk scores of interest changed by including genetic principal components computed with the lower LD thinning threshold ($r^2$=0.2) as model covariates (online supplementary results).

### *1.3. The GENOSCORES platform*

We have developed the GENOSCORES platform which aims to improve genetic prediction by exploiting known genetics of relevant traits such as biomarkers and risk factors. GENOSCORES is a database of published genotype-phenotype associations from well-powered GWAS, accompanied by an R package that computes genotypic scores and performs downstream statistical analyses, including genetic prediction and functional annotation. A flowchart with the analytical workflows currently implemented in GENOSCORES is shown in Figure S1.

The database content was collected during 2016-2017 by mining the literature for well-powered GWAS studies and GWAS meta-analyses that provided access to summary statistics across the whole-genome or with a liberal cut-off threshold ($p$-value $< 10^{-4}$). For GWAS studies performing trans-ethnic analyses, such as the GWAS of RA by [2], we imported summary statistics based on European ancestry samples, when these were made available. Extending the database content with GWAS of non-European and mixed ancestry populations will be sought in future updates. A list of studies available in GENOSCORES when we performed this work is given in Table S1.

### *1.4. GENOSCORES score computation*

Three user-defined parameters are used for the score computation: a $p$-value threshold for filtering SNPs to be included in the scores, a $p$-value threshold for defining trait-associated regions and a genomic distance for defining the boundaries of a trait-associated region. The boundaries of a genomic region are defined by positions at which there is a gap of at least the prespecified distance from any other SNP in the filtered set of SNPs for that GWAS.

In principle, one could find the optimal values for these parameters, for instance by performing a grid search over a set of values and evaluating predictive performance on held-out data. For a robust evaluation, a large sample size is required. Therefore, in this work we used a single setting for these parameters and used the available data to identify genomic regions of interest, rather than optimise the regional scores.

We used $p < 10^{-5}$ for filtering SNPs, $p < 10^{-7}$ for defining trait-associated regions and distance = 1 megabase for defining region boundaries. A $p < 10^{-7}$ is more liberal than the genome-wide significance threshold ($5 \times 10^{-8}$), thus allowing construction of scores for regions with a suggestive signal, but still stringent enough to reduce the number of false positive regions, which can then be handled by the hierarchical shrinkage prior. A $p < 10^{-5}$ allows inclusion of multiple associated SNPs in each region, which typically leads to better "tagging" of the underlying causal locus, while keeping the computational cost of the ajustment for linkage disequilibrium (LD) low. Finally, a distance of 1 megabase is large enough to ensure that SNPs outside the boundaries of a region are independent from SNPs within the region.

Occasionally, two independent signals were grouped in the same regional score. For instance, in the RA regional scores, the *IL2RA* locus was grouped together with the *PRKCQ*, *ARID5B* was grouped together with *RTKN2*, and *MED1* was

grouped together with *IKZF3-CSF3* (Table S2). However, the computation of the regional scores based on the LD-adjustment is equivalent to fitting a multivariate model in each region and thus the resulting score should be represent both signals.

To perform the LD-adjustment, we multiplied the vector of univariate coefficients for SNPs in each region by the inverse of the SNP-SNP correlation matrix. In regions with high LD, the SNP-SNP correlation matrix can be ill-conditioned and thus difficult to invert. To handle such rank deficiencies in the correlation matrix we implemented a pseudo-inverse solution, where we perform an eigendecomposition of the matrix, truncate small and zero eigenvalues and compute the inverse based on the remaining components.

### *1.5. Genome-wide score for RA*

We constructed a genome-wide score for RA by adding the 37 regional scores used in the prediction analyses and any SNPs that passed the filtering $p$-value threshold ($p < 10^{-5}$) but were not assigned to a region. The largest contribution to the genome-wide score is from the regional score at the human leukocyte antigen region (HLA). The correlation between the genome-wide RA score and the HLA-regional RA score was 0.86. We evaluated the univariate association between the genome-wide RA score and the TNFi response outcomes using the full study sample and in stratified analyses.

### *1.6. Filtering of correlated scores*

In multivariate prediction, correlations among genotypic scores resulted in ill-conditioned design matrices in the case of immune cell traits and eQTLs. To avoid numerical instabilities we implemented a filtering step, where we repeatedly computed the singular value decomposition of the design matrix and randomly removed one from each pair of correlated scores until all singular values were greater than $10^{-6}$, starting with an absolute correlation threshold of 0.95 and decreasing by 0.05 at each iteration.

Details of the filtered regional scores we used in multivariate prediction, together with the univariate associations of these scores with all TNFi response outcomes are given in Tables S2, S3, S4 and S5, for RA, immune cell traits, eQTL and mQTL scores, respectively.

## 2. Supplementary Results

### *2.1. Prediction of secondary TNFi response outcomes*

We used change in the two subjective components of the DAS ($\Delta$TJC and $\Delta$GHVAS) and the composite score ($\Delta$DAS28-ESR4) as secondary outcomes quantifying TNFi response. TNFi response as quantified by $\Delta$DAS28-ESR4 improved by including regional genotypic scores for RA risk or eQTL scores of implicated genes in penalised regression (Table S7).

In agreement with prediction results for the primary TNFi response outcomes, the regional score for RA at the *CD40* locus had the highest explanatory power

for $\Delta$DAS28-ESR4. The univariate association of the RA score at the *CD40* locus with $\Delta$DAS28-ESR4 passed the *p*-value threshold corrected for the number of RA scores and five response phenotypes ($p = 0.00012$). The direction of the effect was consistent with that for $\Delta$SJC and $\Delta$ESR, with higher RA load at the *CD40* locus being associated with better TNFi response.

No other score was significantly associated with any of the secondary TNFi response outcomes at Bonferroni correction. Prediction of $\Delta$TJC and $\Delta$GHVAS did not generally improve by including regional genotypic scores in penalised regression models. Note that to facilitate comparison we also included prediction results for the primary TNFi response outcomes ($\Delta$SJC and $\Delta$ESR; also presented in the main manuscript) in Table S7.

### *2.2. Stratification by TNFi agent*

It is possible that response to different TNFi agents is affected by different genetic loci. Patients included in our sample were primarily treated with either adalimumab (n=1255), infliximab (n=792), or etanercept (n=721). The sample size for each individual drug is too small to allow for a complete evaluation of all genotypic scores examined in this study –especially in the absence of strong findings using the full study sample (n=2938). However, we performed an analysis stratified by TNFi agent for the two genotypic scores significantly associated with TNFi response (across all agents) at the Bonferroni-corrected *p*-value threshold (RA score at *CD40* and score for "CD39 on CD 4 T cells" at *ENTPD1*) and the genome-wide RA score.

Table S8 shows univariate associations between genotypic scores and TNFi response outcomes using the full study sample, or groups of patients each receiving a different TNFi agent. For the associations that passed Bonferroni-correction in the original analyses (score for "CD39 on CD 4 T cells" at *ENTPD1* and RA score at *CD40* with $\Delta$SJC), the direction of the effect was consistent among all groups and the confidence intervals for the effect sizes overlapped. The genome-wide RA score was not significantly associated with either TNFi response outcome, in agreement with earlier studies looking at the association between polygenic risk scores for RA and TNFi response [3].

### *2.3. Adjustment for ACPA and smoking status*

Status for anti-citrullinated protein antibodies (ACPA) and for smoking was available for approximately a third of the samples, which prevented us from using them as covariates in the full statistical analyses. However, these covariates have been reported to influence response to treatment in RA, and seropositive and seronegative RA is also likely to be affected by different genetic loci. We therefore tested if the associations of TNFi response with the genotypic scores for "CD39 on CD4 T cells" at the *ENTPD1* locus, RA at the *CD40* locus, and RA genome-wide changed when additionally adjusted for ACPA and smoking status (Table S9).

The estimated effects of the genotypic scores on TNFi response remained consistent when we adjusted for the additional covariates, with a great overlap

between confidence intervals. The $p$-values of association were higher, but this is due to the reduction in sample size. We note a small increase in the effect sizes for the two associations that passed Bonferroni-correction in the original analyses (score for "CD39 on CD 4 T cells" at *ENTPD1* and RA score at *CD40* with ΔSJC).

### *2.4. Adjustment for genetic principal components with different LD-thinning*

We tested if the associations of TNFi response with the genotypic scores for "CD39 on CD4 T cells" at the *ENTPD1* locus, RA at the *CD40* locus and RA at the HLA region changed when adjusting for genetic principal components computed with a lower threshold for LD-thinning ($r^2 = 0.2$). The estimated effects of the genotypic scores on TNFi response did not change by including the revised PCs as covariates in the model (Table S10).

### *2.5. Association with CD73 genotypic score*

Similar to CD39, CD73 is a cell surface ectonucleotidase involved in adenosine production in regulatory T cells. We examined a regional genotypic score for the cell subset frequency of CD73+ cells in the CD4 T cell population. We note that the [4] study did not report any genetic associations with cell surface expression level of CD73. The genotypic score cell subset frequency was based on SNPs at the *NT5E* locus, which codes for CD73, and was correlated with the cis-acting eQTL score for *NT5E* (correlation = 0.87). There was no association of the score for cell subset frequency of CD73 nor the *NT5E* eQTL score with either ΔSJC or ΔESR (Table S11).

## 3. Supplementary References


[1] Chang CC, Chow CC, Tellier LC, Vattikuti S, Purcell SM, Lee JJ. Second-generation PLINK: Rising to the challenge of larger and richer datasets. GigaScience 2015;4:7. doi:10.1186/s13742-015-0047-8.

[2] Okada Y, Wu D, Trynka G, Raj T, Terao C, Ikari K, et al. Genetics of rheumatoid arthritis contributes to biology and drug discovery. Nature 2014;506:376–81. doi:10.1038/nature12873.

[3] Jiang X, Askling J, Saevarsdottir S, Padyukov L, Alfredsson L, Viatte S, et al. A genetic risk score composed of rheumatoid arthritis risk alleles, HLA-DRB1 haplotypes, and response to TNFi therapy – results from a Swedish cohort study. Arthritis Research & Therapy 2016;18. doi:10.1186/s13075-016-1174-z.

[4] Roederer M, Quaye L, Mangino M, Beddall MH, Mahnke Y, Chattopadhyay P, et al. The genetic architecture of the human immune system: A bioresource for autoimmunity and disease pathogenesis. Cell 2015;161:387–403. doi:10.1016/j.cell.2015.02.046.

Table S1: Studies available in the GENOSCORES database at the time of this work. For each study we list the pubmedid, the number of GWAS analyses (different traits), the total number of GWAS coefficients (across traits) and the maximum GWAS $p$-value (i.e. the cut-off threshold for reporting summary statistics).

| |
|---|
| table given in file: [annrheumdis-2018-214877supp002.csv] |

Table S2: Details of RA regional scores and results from univariate association of each score with each TNFi response outcome.

| |
|---|
| table given in file: [annrheumdis-2018-214877supp003.csv] |

Table S3: Details of regional scores for immune cell traits and results from univariate association of each score with each TNFi response outcome.

| |
|---|
| table given in file: [annrheumdis-2018-214877supp004.csv] |

Table S4: Details of regional scores for the expression of implicated genes and results from univariate association of each score with each TNFi response outcome.

| |
|---|
| table given in file: [annrheumdis-2018-214877supp005.csv] |

Table S5: Details of regional scores for the methylation of implicated genes and results from univariate association of each score with each TNFi response outcome.

| |
|---|
| table given in file: [annrheumdis-2018-214877supp006.csv] |

Table S6: Number of samples with complete measurements for each TNFi response outcome and two sets of clinical and lifestyle covariates. Top row (set 1): baseline DAS components, gender, cohort, genotyping array, 10 genetic principal components. Bottom row (set 2): all covariates in set 1, ACPA status, smoking status.

| | ΔDAS28-ESR4 | ΔESR | ΔSJC | ΔTJC | ΔGHVAS |
|---|---|---|---|---|---|
| Set 1 (used in prediction models) | 2825 | 2872 | 2922 | 2921 | 2903 |
| Set 2 (used for additional adjustments) | 899 | 909 | 923 | 923 | 915 |

Table S7: Prediction of primary and secondary TNFi response outcomes using penalised regional genotypic scores for different types of intermediate traits. Prediction performance is quantified by the difference in test log-likelihood (in nats) between a model with clinical covariates and genotypic scores and a model with clinical covariates only, and by the percent of phenotypic variance explained (in parenthesis). Results from 10-fold cross-validation.

| Intermediate trait type | ΔDAS28-ESR4 | ΔESR | ΔSJC | ΔTJC | ΔGHVAS |
|---|---|---|---|---|---|
| Rheumatoid arthritis | 7.0 (0.38%) | -1.6 (0%) | 5.3 (0.26%) | -0.5 (0%) | 2.0 (0.06%) |
| Immune cell traits | -0.6 (0%) | 2.9 (0.17%) | -0.7 (0%) | 0.7 (0.02%) | -0.5 (0%) |
| eQTLs | 2.2 (0.11%) | 2.9 (0.17%) | 3.4 (0.16%) | 1.0 (0.03%) | -1.7 (0%) |
| eQTLs & mQTLs | 1.4 (0.06%) | 1.6 (0.09%) | 2.5 (0.11%) | 0.2 (0%) | 1.2 (0.03%) |

Table S8: Univariate associations between TNFi response outcomes and genotypic scores of interest stratified by TNFi agent. The coefficients are the effect sizes of the standardised score on the standardised phenotype.

| Group | Sample size | CD39 on CD4 T score | | RA score at CD40 | | Genome-wide RA score | |
|---|---|---|---|---|---|---|---|
| | | Coefficient (St.Error) | $p$-value | Coefficient (St.Error) | $p$-value | Coefficient (St.Error) | $p$-value |
| **Change in SJC** | | | | | | | |
| All Samples | 2922 | -0.07 (0.02) | 5e-05 | 0.07 (0.02) | 0.0004 | 0.01 (0.02) | 0.5 |
| Adalimumab | 1248 | -0.06 (0.03) | 0.02 | 0.06 (0.03) | 0.02 | 0.02 (0.03) | 0.3 |
| Infliximab | 787 | -0.1 (0.04) | 0.002 | 0.08 (0.04) | 0.04 | 0.01 (0.04) | 0.8 |
| Etanercept | 718 | -0.07 (0.04) | 0.05 | 0.06 (0.04) | 0.09 | 0.02 (0.04) | 0.5 |
| **Change in ESR** | | | | | | | |
| All Samples | 2872 | -0.003 (0.02) | 0.9 | 0.05 (0.02) | 0.01 | 0.04 (0.02) | 0.05 |
| Adalimumab | 1230 | -0.04 (0.03) | 0.1 | 0.03 (0.03) | 0.2 | 0.005 (0.03) | 0.8 |
| Infliximab | 781 | 0.04 (0.04) | 0.2 | 0.06 (0.04) | 0.1 | 0.09 (0.04) | 0.02 |
| Etanercept | 696 | 0.02 (0.04) | 0.5 | 0.05 (0.04) | 0.1 | 0.05 (0.04) | 0.2 |

Table S9: Univariate associations between genotypic scores of interest and TNFi response outcomes adjusted by different sets of covariates. Set 1: baseline DAS components, gender, cohort, genotyping array, 10 genetic principal components. Set 2: all covariates in set 1, ACPA status, smoking status. The coefficients are the effect sizes of the standardised score on the standardised phenotype after adjusting for covariates.

| Covariates | Sample size | CD39 on CD4 T score | | RA score at CD40 | | Genome-wide RA score | |
|---|---|---|---|---|---|---|---|
| | | Coefficient (St.Error) | $p$-value | Coefficient (St.Error) | $p$-value | Coefficient (St.Error) | $p$-value |
| **Change in SJC** | | | | | | | |
| Set 1 | 2922 | -0.07 (0.02) | 5e-05 | 0.07 (0.02) | 0.0004 | 0.01 (0.02) | 0.5 |
| Set 2 | 903 | -0.1 (0.03) | 0.003 | 0.08 (0.03) | 0.02 | 0.004 (0.03) | 0.9 |
| **Change in ESR** | | | | | | | |
| Set 1 | 2872 | -0.003 (0.02) | 0.9 | 0.05 (0.02) | 0.01 | 0.04 (0.02) | 0.05 |
| Set 2 | 889 | -0.03 (0.03) | 0.4 | 0.02 (0.03) | 0.5 | 0.05 (0.03) | 0.2 |

Table S10: Univariate associations between genotypic scores of interest and TNFi response outcomes adjusted by genetic principal components computed with different thresholds for LD-thinning. The coefficients are the effect sizes of the standardised score on the standardised phenotype after adjusting for principal components and clinical covariates.

| LD-thin $r^2$ | Sample size | CD39 on CD4 T score | | RA score at CD40 | | RA score at HLA | |
|---|---|---|---|---|---|---|---|
| | | Coefficient (St.Error) | $p$-value | Coefficient (St.Error) | $p$-value | Coefficient (St.Error) | $p$-value |
| **Change in SJC** | | | | | | | |
| 0.9 | 2922 | -0.07 (0.02) | 5e-05 | 0.07 (0.02) | 0.0004 | 0.03 (0.02) | 0.1 |
| 0.2 | 2922 | -0.08 (0.02) | 4e-05 | 0.06 (0.02) | 0.0006 | 0.03 (0.02) | 0.1 |
| **Change in ESR** | | | | | | | |
| 0.9 | 2872 | -0.003 (0.02) | 0.9 | 0.05 (0.02) | 0.01 | 0.04 (0.02) | 0.04 |
| 0.2 | 2872 | -0.004 (0.02) | 0.8 | 0.05 (0.02) | 0.01 | 0.04 (0.02) | 0.06 |

Table S11: Univariate associations between TNFi response outcomes phenotypes and genotypic scores at the *NT5E* locus. The coefficients are the effect sizes of the standardised score on the standardised phenotype.

| Response phenotype | Genetic score | Coefficient | $p$-value |
|---|---|---|---|
| ΔSJC | CD4:%Treg(73+) | 0.02 | 0.3 |
| ΔSJC | *NT5E* eQTL | 0.007 | 0.7 |
| ΔESR | CD4:%Treg(73+) | -0.03 | 0.1 |
| ΔESR | *NT5E* eQTL | -0.02 | 0.3 |

Table S12: MATURA collaborators and affiliations.

| table given in file: [annrheumdis-2018-214877supp007.docx] |
|---|

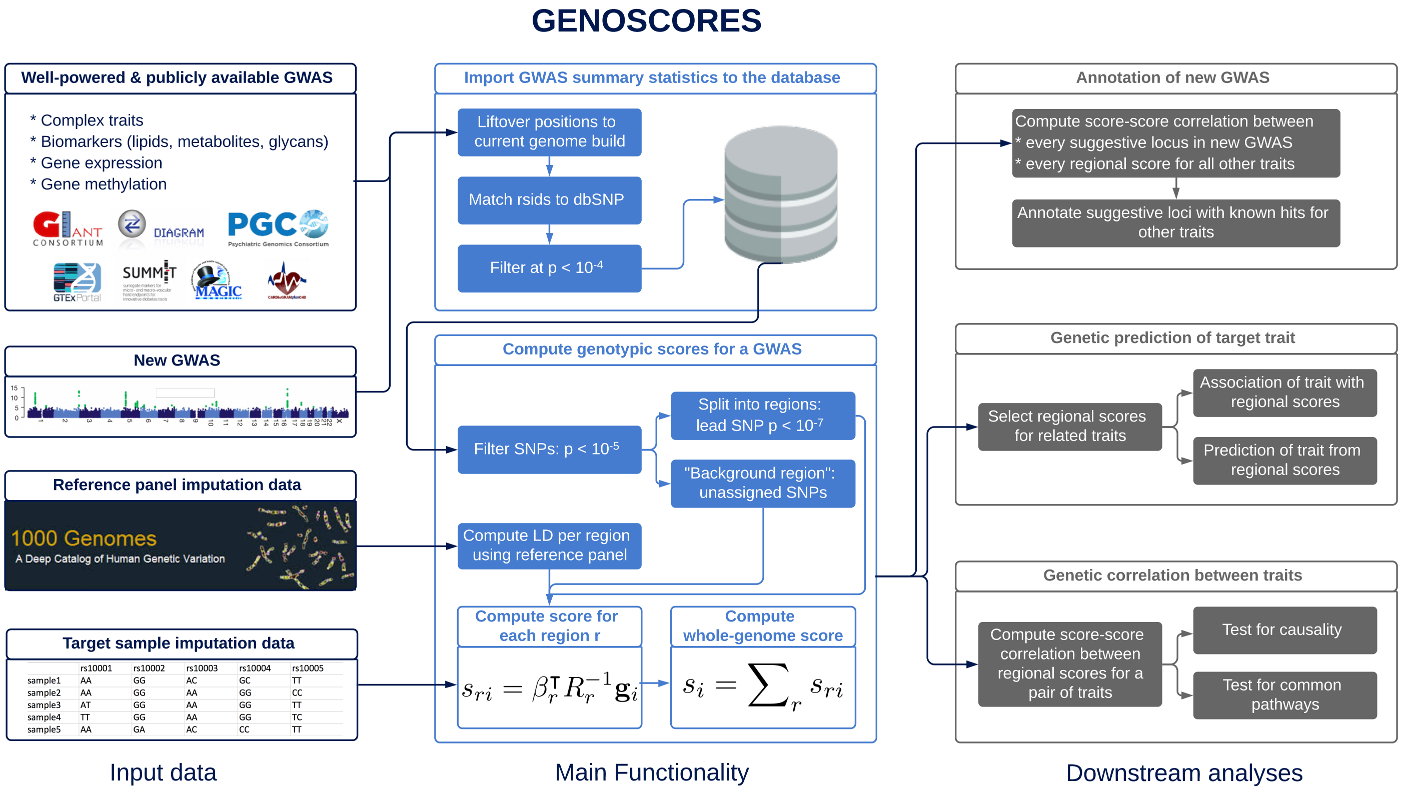


Figure S1: GENOSCORES flowchart demonstrating inputs, functionality and outputs.

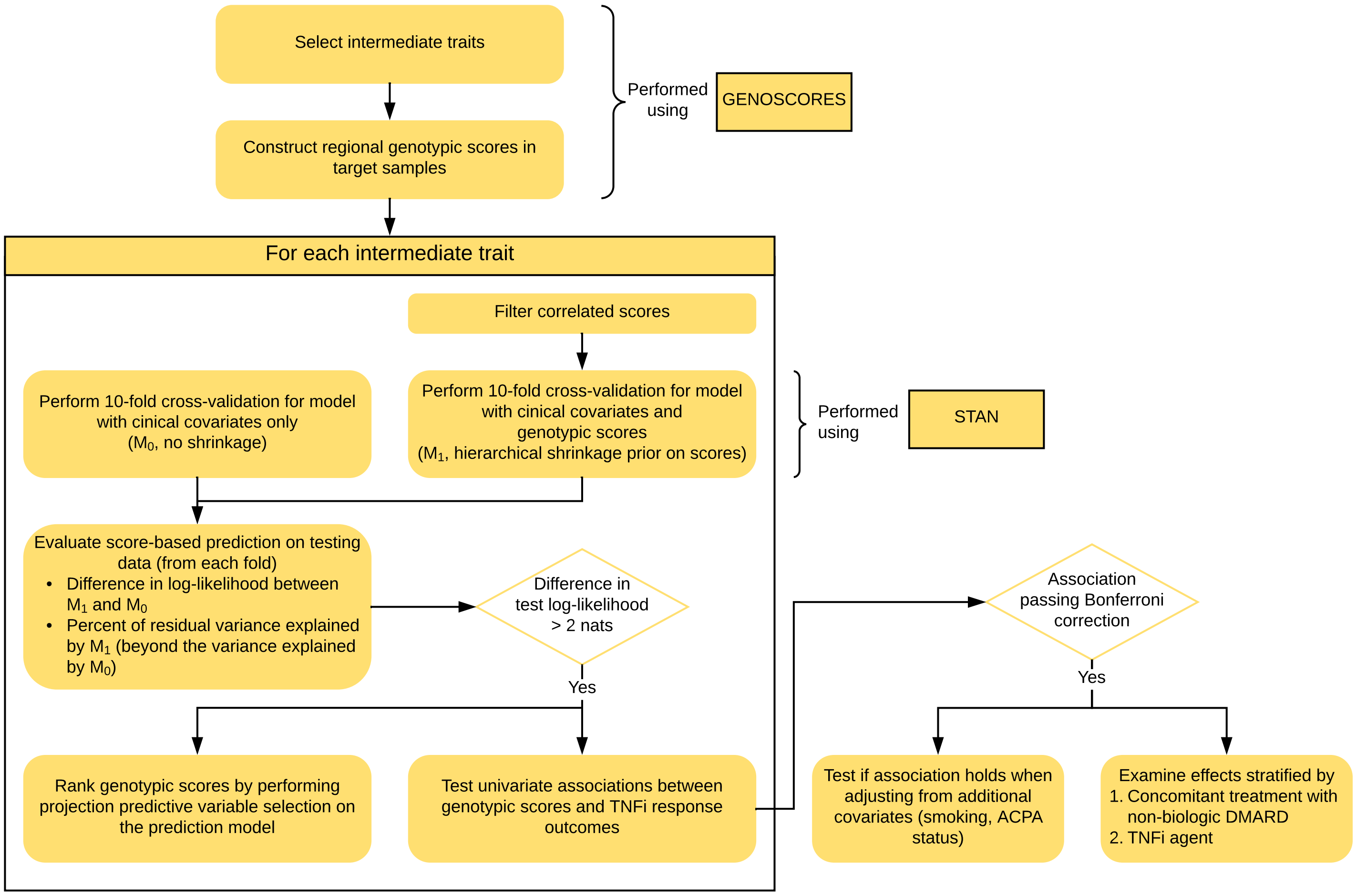


Figure S2: Diagram of the statistical analysis pipeline.
