# Supplementary material for: Association of response to TNF inhibitors in rheumatoid arthritis with quantitative trait loci for CD40 and CD39
Source: Ann Rheum Dis. 2019 Apr 29;78(8):1055–61. doi: 10.1136/annrheumdis-2018-214877 (PMC6669378; doi:10.1136/annrheumdis-2018-214877)
Supplement: Supplementary data [file annrheumdis-2018-214877supp007.docx]

| **Name** | **Institution** | **Email contact** |
| --- | --- | --- |
| **Work Stream 1** |  |  |
| Prof Costantino Pitzalis | Queen Mary University of London | c.pitzalis@qmul.ac.uk |
| Prof Peter Taylor | University of Oxford | [peter.taylor@kennedy.ox.ac.uk](mailto:peter.taylor@kennedy.ox.ac.uk) |
| Prof Ernest Choy | Cardiff University | [ChoyEH@cardiff.ac.uk](mailto:ChoyEH@cardiff.ac.uk) |
| Prof Iain McInnes | University of Glasgow | [Iain.McInnes@glasgow.ac.uk](mailto:Iain.McInnes@glasgow.ac.uk) |
| Dr Mike Barnes | Queen Mary University of London | [m.r.barnes@qmul.ac.uk](mailto:m.r.barnes@qmul.ac.uk) |
| Prof John Isaacs | Newcastle University | [J.D.Isaacs@newcastle.ac.uk](mailto:J.D.Isaacs@newcastle.ac.uk) |
| Prof Christopher Buckley | University of Birmingham | [c.d.buckley@bham.ac.uk](mailto:c.d.buckley@bham.ac.uk) |
| Prof Michael Ehrenstein | University College London | [m.ehrenstein@ucl.ac.uk](mailto:m.ehrenstein@ucl.ac.uk) |
| Prof Peter Sasieni | Queen Mary University of London | [p.sasieni@qmul.ac.uk](mailto:p.sasieni@qmul.ac.uk) |
|  |  |  |
|  |  |  |
| **Work Stream 2** |  |  |
| Prof Anne Barton | University of Manchester | [Anne.Barton@manchester.ac.uk](mailto:Anne.Barton@manchester.ac.uk) |
| Prof Ann Morgan | University of Leeds | [mrpawm@leeds.ac.uk](mailto:mrpawm@leeds.ac.uk) |
| Prof Gerry Wilson | University College Dublin | [gerry.wilson@ucd.ie](mailto:gerry.wilson@ucd.ie) |
| Prof Paul McKeigue | University of Edinburgh | [paul.mckeigue@ed.ac.uk](mailto:paul.mckeigue@ed.ac.uk) |
| Prof Heather Cordell | Newcastle University | [heather.cordell@newcastle.ac.uk](mailto:heather.cordell@newcastle.ac.uk) |
| Prof Jenny Barrett | University of Leeds | [j.h.barrett@leeds.ac.uk](mailto:j.h.barrett@leeds.ac.uk) |
| Prof Andrew Cope | Kings College London | [andrew.cope@kcl.ac.uk](mailto:andrew.cope@kcl.ac.uk) |
| Prof Adam Young | University of Hertfordshire | [adam.young@nhs.net](mailto:adam.young@nhs.net) |
| Prof Karim Raza | University of Birmingham | [K.Raza@bham.ac.uk](mailto:K.Raza@bham.ac.uk) |
| Prof Katherine Payne | University of Manchester | [Katherine.Payne@manchester.ac.uk](mailto:Katherine.Payne@manchester.ac.uk) |
| Prof Jane Worthington | University of Manchester | [jane.worthington@manchester.ac.uk](mailto:jane.worthington@manchester.ac.uk) |
| Prof Deborah Symmons | University of Manchester | [deborah.symmons@manchester.ac.uk](mailto:deborah.symmons@manchester.ac.uk) |
| Prof Kimme Hyrich | University of Manchester | [Kimme.Hyrich@manchester.ac.uk](mailto:Kimme.Hyrich@manchester.ac.uk) |
| Prof Ian Bruce | University of Manchester | [ian.bruce@manchester.ac.uk](mailto:ian.bruce@manchester.ac.uk) |
|  |  |  |
| **Industry** |  |  |
| Martin Hodge | Pfizer | Martin.Hodge@pfizer.com |
| Anthony Rowe | Janssen | [arowe4@ITS.JNJ.com](mailto:arowe4@ITS.JNJ.com) |
| Jeffrey Siegel | Roche/Genentech | [siegel.jeffrey@gene.com](mailto:siegel.jeffrey@gene.com) |
| Michelle Mao | BGI | [Qian.Mao@bgitechsolutions.com](mailto:Qian.Mao@bgitechsolutions.com) |
| Richard Watts | Qiagen | [Richard.Watts@qiagen.com](mailto:Richard.Watts@qiagen.com) |
| Carolyn Cuff | Abbvie | [carolyn.cuff@abbvie.com](mailto:carolyn.cuff@abbvie.com) |
| David Close | MedImmune | [closeda@MedImmune.com](mailto:closeda@MedImmune.com) |
| Philippe Coterell | Avacta | [philippe.cotrel@avacta.com](mailto:philippe.cotrel@avacta.com) |
| Felix Agakov | Pharmatics | [felix@pharmaticsltd.com](mailto:felix@pharmaticsltd.com) |


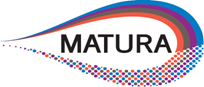
**MATURA Collaborators**
